# Effect of Diet Supplementation with the Mycotoxin Binder Montmorillonite on the Bioavailability of Vitamins in Dairy Cows

**DOI:** 10.3390/toxins14010026

**Published:** 2022-01-01

**Authors:** Abdelhacib Kihal, Cristina Marquès, María Rodríguez-Prado, Eduard Jose-Cunilleras, Sergio Calsamiglia

**Affiliations:** 1 Animal Nutrition and Welfare Service, Departament de Ciència Animal i dels Aliments, Universitat Autònoma de Barcelona, 08193 Bellaterra, Spain; abdelhacib.khl@gmail.com (A.K.); cristina.marques.fabregas@gmail.com (C.M.); Maria.Rodriguez.Prado@uab.cat (M.R.-P.); 2 Equine Internal Medicine Service, Departament de Medicina i Cirurgia Animals, Universitat Autònoma de Barcelona, 08193 Bellaterra, Spain; Eduard.Jose.Cunilleras@uab.cat

**Keywords:** mycotoxin binder, adsorption, bioavailability, vitamins, binding capacity

## Abstract

The objective of this study was to determine the effect of the mycotoxin binder montmorillonite (MMT) supplemented in the diet of dairy cows on the bioavailability of vitamins A, D, E, B1 and B6. Six multiparous Holstein-Friesian cows were used in a crossover design with two periods. Treatments were a control diet with or without MMT. Vitamins were infused individually into the abomasum through the ruminal cannula. Blood samples were collected from the jugular vein at 0, 1, 2, 3, 4, 6, 9, 12, 24 and 48 h after the administration of each vitamin. Results showed that vitamin A reached maximal concentration (Tmax) at 5.3 h after dosing, the maximal concentration (Cmax) was 1.2 times higher than the basal concentration (Cbasal), and the area under the curve (AUC) was 739 arbitrary units. Vitamin B6 reached the Tmax at 13 h after dosing, the Cmax was 1.4 times higher than the Cbasal, and the AUC was 222 arbitrary units. No differences were observed in Cbasal, Tmax, Cmax and AUC of vitamin A and B6 between control vs. MMT-supplemented cows. Plasma concentrations of vitamins D, E and B1 had no concentration peaks, and were not affected by MMT addition. The lack of a response suggests that their plasma concentration may be tightly regulated. Results of this study do not show evidence that MMT affects the bioavailability of vitamins A and B6 in vivo.

## 1. Introduction

Mycotoxin contamination of feeds is common, and a recent survey revealed that 88% of feed samples analyzed from 100 countries contained at least one mycotoxin [1]. Forages grown on dairy farms are also often contaminated and contribute to the mycotoxin load in dairy cattle. Mycotoxin binders (MTB) have been effective in reducing mycotoxin toxicity [2,3], and a recent study reported that 24% of dairy farms in the US use MTB [4]. In a preliminary in vitro study six different MTB were tested for their capacity to bind vitamins and amino acids [5,6], and montmorillonite (MMT) had the highest adsorption capacity. Montmorillonite is a clay-based MTB that binds mycotoxins through weak and unspecific ion-dipole forces with up to 70 to 90% effectiveness [7,8]. However, this unspecific mechanism of action may also sequester other nutrients like some proteins, amino acid (AA) and vitamins [5,6,9]. Therefore, the European Authority for Food Safety [10] requires for approval that all MTB prove that they do not adsorb these essential nutrients. These tests are normally conducted in in vitro conditions that simulate the gastric and intestinal digestion [8]. However, in vitro methods have not been validated in vivo. Therefore, we hypothesized that MMT will adsorb vitamins A, D, E, B1 and B6 in dairy cattle in vivo. 

The aim of this trial was to determine the effect of MMT supplemented in the diet of dairy cows on the bioavailability of vitamins A, D, E, B1 and B6.

## 2. Results

In the current study, the area under the curve (AUC) technique was used to evaluate the effect of MMT on the bioavailability of vitamins A, D, E, B1 and B6 after the administration of a single dose. Plasma concentration of blood samples collected during the 48 h after dosing were fitted to a curve to determine the vitamin kinetics with and without MMT. 

**Figure 1:**
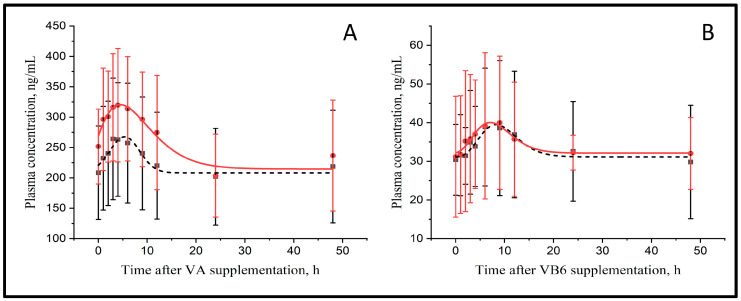
Plasma kinetics of vitamin A (**A**) and vitamin B6 (**B**) in control (■) or after montmorillonite supplementation (●) (*n* = 6).

The parameters that describe the plasma kinetics of vitamins A and B6 are presented in Figure 1 and Table 1. The basal concentration (Cbasal) of vitamin A was similar between treatments (221 vs. 272 ng/mL for CTR and MMT, respectively) and peaked at 5.3 vs. 5.4 h with a 1.23 vs. 1.19-fold increase in maximal concentration (Cmax) compared with Cbasal for CTR and MMT, respectively. Although the curves for CTR and MMT were parallel and separated by about 50 ng/mL, differences were not significant. The AUC that reflects the relative bioavailability of vitamin A (794 vs. 683 arbitrary units, AU, for CTR and MMT, respectively) was not affected by MMT. 

Plasma vitamin B6 concentration increased in response to the abomasal infusion in CTR and MMT cows (Figure 1B). The Cbasal of vitamin B6 was similar between treatments (29.0 vs. 32.7 ng/mL, for CTR and MMT, respectively) and peaked at 11.6 vs. 15.0 h, being Cmax 1.51 vs. 1.32-fold higher than the Cbasal in CTR and MMT, respectively. The AUC was 218 vs. 224 AU for CTR and MMT, respectively, however none of these differences were significant. 

The Cbasal for vitamins D (64.5 vs. 68.5 ng/mL for CTR and MMT, respectively) and E (5.4 vs. 4.9 µg/mL for CTR and MMT, respectively) were similar between treatments. As the curves were flat, the determination of Cmax, time at maximal concentration (Tmax) and the AUC was not possible and, therefore, the effect of MMT on their bioavailability could not be determined. Plasma vitamin B1 concentrations were not affected by the abomasal infusion of the vitamin, with a Cbasal of 26.3 vs. 32.0 ng/mL for CTR and MMT, respectively, resulting in a flat curve where the Cmax, Tmax and the AUC could not be determined. The MMT had no effects on vitamin B1 plasma concentrations.

## 3. Discussion

The potential interaction between MTB and vitamins is associated with the physicochemical characteristics of the different molecules including molecular structure, size, and the capacity to establish weak chemical bonds [9,11]. The molecular weight of vitamins A, D, E and B1 (286, 384, 430 and 265 g/mol, respectively) is similar to most mycotoxins (312, 296, 318 and 312 g/mol for aflatoxin, vomitoxin, zearalenone and nivalenol, respectively). The molecular weight of vitamin B6 (169 g/mol) is smaller, yet contains three hydroxyl groups that may also establish weak bonds. Therefore, it is reasonable to hypothesize that these vitamins may be adsorbed by MTB. Previous in vitro studies demonstrated that many MTB sequestered some vitamins [5,6,9]. Montmorillonite was reported to have the highest adsorption of vitamins B1 (95%) and B6 (63%), and was among the highest for vitamin E (46%) compared with other inorganic and organic MTB evaluated in vitro [5]. However, few studies have shown the impact of the interactions between MTB and vitamins in vivo. The purpose of the current study was to determine the short-term adsorption interactions of MMT with vitamins A, D, E, B1 and B6 on dairy cows by studying their effect on plasma vitamin kinetic curves. The AUC and plasma kinetics methods are a good approach to determine the relative bioavailability of nutrients and have been used previously to study AA bioavailability [12,13]. However, the AUC technique requires overdosing of substrates to generate a well-defined peak on the fitted curves. Kihal et al. [13] used the AUC method to report that supplementing eight times the required dose of AA resulted in a 1.80-fold increase in plasma peak concentrations. In the current study, we used the MMT dose recommended for MTB [2] to effectively bind mycotoxins, but this dose was, on average, eight times the field recommendation to prevent mycotoxin toxicity. As the mechanism of binding is based on ion exchange, maintaining the ratio MTB:vitamin was important as the binding capacity may be saturated and affect results. Therefore, the doses selected for each vitamin were also eight times the recommended levels [14,15], and we expected that such a dose in CTR would allow us to observe a peak concentration after absorption. 

Plasma concentration of vitamin A had a clear curve where the Cbasal (average of 250 ng/mL) was consistent with the Cbasal reported for dairy cows, ranging from 184 to 336 ng/mL [16]. Results allowed us to calculate the Tmax, Cmax and the AUC after a single dose, however the kinetic parameters and the AUC were not affected by the supplementation of MMT. There are no similar studies where plasma vitamin A concentration kinetics or the possible adsorption by MTB have been studied in dairy cattle after a single-dose administration. However, the lack of effect of MMT on vitamin A bioavailability agrees with [17] who reported no effects of clinoptilolite, another clay-based MTB, on vitamin A bioavailability during the entire lactation in dairy cows. Other studies in chicks also reported no effects of bentonite, another clay-based mycotoxin binder, on liver concentration of vitamin A as an indicator of its bioavailability [18,19]. Therefore, there seems to be no evidence that MTB in general, and MMT in particular, interfere with the bioavailability of vitamin A in vivo. 

The concentration of vitamin B6, like vitamin A, showed a clear curve. Results allowed us to calculate the Cbasal, Tmax, the Cmax and the AUC after a single dose, however the kinetic parameters and the AUC were not affected by the inclusion of MMT. The absorption of vitamin B6 in the intestine occurs by passive diffusion. For this reason, it is surprising that the Tmax (average of 13.3 h) was so late compared with vitamin A or some AA [8]. Plasma vitamin B6 concentrations returned to the Cbasal after 24 h and the AUC of the 2 curves (average of 221 AU) was similar between treatments, suggesting that MMT did not interact with vitamin B6 bioavailability. We are not aware of other in vivo studies on the interaction between MTB and vitamin B6 in cattle. However, in vitro studies reported that vitamin B6 adsorption was high and up to 72 and 98% in MMT [8,20]. The inconsistencies between the high binding capacity in vitro compared with the lack of evidence for binding in vivo requires further research, and in vitro methods currently in use need to be validated.

The Cbasal of 25-OH-vitamin D (average of 66.6 ng/mL), E (average of 5.1 µg/mL) and B1 (average of 29.2 ng/mL) were similar between treatments, and consistent with the Cbasal in dairy cows that ranged from 40 to 100 ng/mL for 25-OH-vitamin D [21], from 4.01 to 6.41 µg/mL for vitamin E [22] and from 14.7 to 34.9 ng/mL for vitamin B1 [23]. However, plasma kinetics response to the infused dose was different from that of vitamin A and B6, where no changes were observed after supplementing a dose eight times higher than physiological conditions. This lack of effect may be attributed to either a tight metabolic regulation of its plasma concentration, or the use of a dose too low to elicit a change in plasma concentration. Poindexter et al. [24] suggested that the increase in plasma 25(OH)D_3_ concentration has a negative feedback on the 25-hydroxylase in the liver to maintain the basal levels of 25-OH-vitamin D constant, which demonstrate a homeostatic regulation that may explain the lack of changes in plasma 25-OH-vitamin D concentration. Hymøller et al. [22] suggested that the absorption of vitamin E in the intestine is the major limiting step and is dependent on dietary fat content. Vitamin B1 is absorbed through an active-saturable and Na^+^ dependent mechanism, which may control its absorption [25]. If the plasma concentration is tightly regulated, then other markers may be required to evaluate the impact of MTB on the bioavailability of these vitamins.

In contrast, Hymøller et al. [22] reported that a single dose of 250 mg of vitamin D resulted in a twofold increase in peak concentration 30 h after dosing, however the dose was 60 times higher than in our study. Hymøller et al. [22] also reported only a small increase in plasma vitamin E (+0.5 µg/mL) after the administration of all-rac-α-Tocopherol acetate at 4.4 g/d, a dose 27 times higher than the amount infused in the current experiment. In contrast, Hidiroglou et al. [26] reported that the oral supply of 12.5 g/cow of all-rac-α-Tocopherol reached a Cmax of 10.8 µg/mL at a Tmax of 35 h and AUC of 1995 AU. Again, this curve was achieved at a dose 78 times higher than the one used in this experiment. Unfortunately, we are not aware of other in vivo studies testing the effects of infusing a high dose of vitamin B1 on changes in plasma vitamin B1 concentrations in dairy cattle. These previous data suggest that the dose of vitamin D and E used in the current experiment may have been too small to create a peak concentration. However, as discussed previously, the mode of action of MMT to adsorb mycotoxins or other essential nutrients is based on an ion-dipole interaction in binding sites. As the reaction may be saturated, it was important for the objective of this experiment to maintain an MMT:vitamin ratio close to physiological conditions. Therefore, using doses high enough to elicit a curve would most likely have saturated the binding capacity of the MMT, confounding results. Alternatively, we could also have fed doses of MMT above 60–75 times higher than recommended levels although it would be unfeasible in vivo. Regardless of the justification for the lack of response in plasma vitamin concentrations, the kinetic parameters of vitamins D, E and B1, and the AUC, could not be calculated. Therefore, the hypothesis that MMT binds vitamins D, E and B1 in dairy cattle under normal feeding conditions could not be confirmed. 

Previous in vitro studies reported that MTB had a small binding capacity towards vitamin D [6,7], and no in vivo data are available to support these findings. The impact of MTB on vitamin E availability is controversial. In vivo, Katsoulos et al. [17] reported no effect of clinoptilolite, a clay-based MTB, on vitamin E bioavailability along the entire lactation period. Other studies in chicks also reported no effect of clinoptilolite and bentonite on vitamin E absorption and its content in the liver [27,28]. In contrast, in vitro studies have shown some variability, with 30% adsorption of vitamin E by MMT [6] or no absorption by bentonite [7]. Data on vitamin B1 is even more limited. In vitro studies demonstrated that MTB, in general, and MMT in particular, adsorbed up to 90% of vitamin B1 [8,29]. An in vivo study on chicks demonstrated that dietary supplementation with MTB reduced vitamin B1 content in the liver [27]. Unfortunately, no other in vivo data is available in cattle and results from this in vivo research do not allow us to provide additional data on the interaction between MMT and vitamin B1.

## 4. Conclusions

Results indicate that MMT does not compromise the bioavailability of vitamins A and B6 in vivo. Results for vitamins D, E and B1 do not allow us to confirm the hypothesis that MMT binds these vitamins. These results are in contrast with previous in vitro studies using the same substrates and, therefore, the use of in vitro tests that simulate the gastro-intestinal tract of animals may not reflect the true values of the capacity of MTB to adsorb vitamins in vivo.

## 5. Materials and Methods

### 5.1. Animals and Diet

The study was conducted at the Farm and Field Experimental Service of the Universitat Autònoma de Barcelona following the protocol approved by the Human and Animal Research Ethics Committee of the University (CEEAH, Barcelona, Spain, protocol # 4652).

Six multiparous, non-pregnant Holstein–Friesian cows (640 ± 40 kg, 32 kg/d of milk and 175 days postpartum) were housed individually in a tie-stall barn with a rubber mat floor. Cows were surgically equipped with a 10-cm rumen cannula (Bar Diamond Inc., Parma, ID, USA), and an infusion line inserted through the rumen cannula into the abomasum [30]. Cows were fed a total mixed diet ad libitum, offered twice a day at 09:00 and 17:00, and with free access to water. Cows were milked twice a day at 07:30 and 17:30, and had 2 h/d of exercise in an open outdoor lot with free access to water, although not to feed. The diet was formulated to meet the requirements of a Holstein cow producing 32 kg of milk/d using the CNCPS program (v6.5, Cornell University, Ithaca, NY, USA). The diet consisted (in dry matter basis) of 44% alfalfa hay, 28% corn grain ground fine, 7% solvent soybean meal, 6% dry beet pulp, 4% cottonseed, 4% beet molasses, 4% of a vitamin/mineral mix, 1.5% fat (Magnapac^®^, Norel, Barcelona, Spain), 1% sodium bicarbonate and 0.5% urea. Diet contained (in dry matter basis) 2.42 Mcal ME/kg, 16.8% crude protein, 29.0% neutral detergent fiber, 18.2% acid detergent fiber, 38.4% non-fibrous carbohydrates, 23.0% starch, 4.6% ether extract and 11.2% ash. 

### 5.2. Experimental Design and Sample Collection 

The experiment was designed as a crossover with two periods. In period one, cows were randomly assigned into two groups, CTR and MMT. Control cows were fed the total mixed diet, and the MMT cows were fed the same total mixed diet plus 1.2% dry matter basis of MMT following the recommendations of Diaz et al. [2]. The MMT (Smectagri^®^, Adiel, Loudeac, France) was mixed with the total mixed diet each morning during the adaptation and treatment periods. Montmorillonite was selected based on a previous in vitro study where it had the highest capacity to adsorb vitamins compared with other organic and inorganic binders [5,6]. Vitamin treatments were: 160 mg/cow of vitamin A (retinyl palmitate, PHR1235, Sigma-Aldrich, St. Louis, MO, USA), 4 mg/cow of vitamin D (cholecalciferol, C9756, Sigma-Aldrich), 160 mg/cow of vitamin E (DL-all-rac-α-Tocopherol, T3376, Sigma-Aldrich), 240 mg/cow of vitamin B1 (thiamine, T4625, Sigma-Aldrich) and 240 mg/cow of vitamin B6 (pyridoxine, P5669, Sigma-Aldrich). The doses of each vitamin were based on the National Research Council Recommendations for dairy cattle [14] and updated by Barroeta et al. [15]. Each period consisted of a 7-d adaptation to the MMT, after which vitamins were dosed individually every other day at 08:00. Vitamin solutions were prepared, dissolved in ethanol for fat-soluble vitamins A, D and E; and in water for water-soluble vitamins B1 and B6. From each solution, 10 mL containing the appropriate dose of each vitamin were infused into the abomasum. Vitamins were prepared daily into opaque flasks to minimize exposure to air and light in the morning of the infusion day and prevent any possible degradation over time. After all vitamins were infused, there was a 2-d washout period to minimize carryover effects within a period, and cows switched treatments for period two, which followed the same protocol.

Blood samples were collected through an indwelling catheter (Angiocath^™^ 14 Gauge 5.25 Inch, 2.1 × 133 mm, Becton Dickinson, Ciudad de México, Mexico) implanted in the jugular vein using an aseptic technique and secured by skin suture under local anesthesia one day before the sampling period. Samples were collected immediately before infusion (0 h) and at 1, 2, 3, 4, 6, 9, 12, 24 and 48 h after abomasal infusion. Blood was collected directly into 6 mL EDTA-coated vacutainer^®^ tubes (K2E, BD-Plymouth, UK) for vitamins B1 and B6, and into 6 mL serum vacutainers^®^ tubes (clot activator tube, BD-Plymouth, PL6 7BP, UK) for vitamins A, D and E. After each sampling, 5 mL of heparinized isotonic saline (0.9% NaCl, 10 IU of heparin/mL) were flushed into the indwelling catheters to prevent blood clotting. Samples were immediately centrifuged at 1000× *g* for 10 min at room temperature in darkness to extract serum for vitamins A, D and E, or plasma for vitamin B6 to minimize light degradation. Blood samples from vitamin B1 treatment were processed without centrifugation. Samples were divided in 2 mL aliquots, preserved in amber vials for maximum protection from light, and frozen at −80 °C for further analysis. Vitamins A, E, B1 and B6 were analyzed using an Ultra-HPLC standard method (Laboratorio Echevarne, Barcelona, Spain), and plasma 25-OH-Vitamin D was analyzed by HPLC-UV standard method (Laboratory of Pharmacology, Universitat Autonoma de Barcelona, Barcelona, Spain).

### 5.3. Curve Fitting and Statistical Analysis

The kinetic values of the vitamin concentration of all vitamins after their infusion were modeled using regression curve fitting software (OriginLab 2000, OriginLab Corporation, Northampton, MA, USA, SPSS Inc., Chicago, IL, USA). Plasma vitamin A and B6 kinetics were adjusted to nonlinear regression models that resulted in the lower Akaike information criterion value and the Cbasal, Tmax, Cmax and the AUC were calculated. Data from vitamin kinetics were analyzed according to a crossover design using the PROC MIXED procedure of SAS (v 9.4, SAS Inst. Inc., Cary, NC, USA). The assumptions of normality and homogeneity of variance were checked graphically using histograms, normal quantile plots and plots of residuals versus fitted predicted values. The linear mixed model used was Yijk = μ + Ti + Sj + Ck + εijk, where: Yijk = variable responses (basal concentration, Cmax, Tmax and AUC); μ = overall mean, Ti = fixed effect of treatment (I = CTR or MMT), Sj = fixed effect of sequence, Ck = random effect of cow, and εijk = residual errors. The results are reported as the least square means and associated standard errors. Statistical differences were declared at *p* ≤ 0.05.

## Figures and Tables

**Table 1:** Plasma kinetic characteristics of vitamins A and B6 after a single dose infusion to dairy cows fed a control (CTR) or montmorillonite (MMT) supplementation (*n* = 6).

Item	Vitamin A				Vitamin B6			
	CTR	MMT	SEM ^1^	P	CTR	MMT	SEM ^1^	P
C_basal_^2^, ng/mL	221	272	33.4	0.20	29.0	32.7	4.3	0.6
C_max_^3^, ng/mL	273	325	36.8	0.32	43.8	43.4	5.9	0.9
T_max_^4^, h	5.3	5.4	0.75	0.89	11.6	15.0	3.1	0.5
AUC ^5^, AU	794	683	228	0.67	218	224	64.5	0.95

^1^ SEM: Standard error of the mean. ^2^ Cbasal: basal concentration at t = 0 h. ^3^ Cmax: maximal concentration. ^4^ Tmax: time at Cmax. ^5^ AUC: area under the curve; AU, arbitrary units.

## Data Availability

The data presented in this study are available on request from the corresponding author.
